# The emerging role of circadian rhythms in the development and function of thermogenic fat

**DOI:** 10.3389/fendo.2023.1175845

**Published:** 2023-05-24

**Authors:** Xuemin Peng, Yong Chen

**Affiliations:** ^1^ Division of Endocrinology, Internal Medicine, Tongji Hospital, Huazhong University of Science and Technology, Wuhan, China; ^2^ Laboratory of Endocrinology, Tongji Hospital, Huazhong University of Science and Technology, Wuhan, China; ^3^ Branch of National Clinical Research Center for Metabolic Diseases, Hubei, China

**Keywords:** brown/beige fat, circadian rhythms, obesity, thermogenesis, circadian clock

## Abstract

Circadian rhythms regulate many biological processes in response to ambient influences. A disrupted circadian rhythm has been shown to be associated with obesity and obesity-related metabolic disorders. Thermogenic fat, including brown and beige fat, may play an important role in this process since it displays a high capacity to burn fat and release the stored energy as heat, contributing to the combat against obesity and its associated metabolic disorders. In this review, we summarize the relationship between the circadian clock and thermogenic fat and the prominent mechanisms which are involved in the regulation of the development and function of thermogenic fat by circadian rhythms, which may provide novel therapeutics for the prevention and treatment of metabolic diseases by targeting thermogenic fat in a circadian manner.

## Introduction

Almost every organism exhibits a circadian rhythm in adaptation to environmental changes. These circadian rhythms are self-sustained by an endogenous timekeeping system which is also called circadian clock. The circadian clocks consist of the central circadian clock in the suprachiasmatic nuclei (SCN) and the peripheral clocks in peripheral tissues (1). SCN receives photic information (light signals) from the retina and then regulates the peripheral clocks to coordinate circadian outputs. In addition, other nonphotic signals, such as food, exercise, sleep, and temperature, can also regulate circadian rhythms by central and peripheral clocks (2). Many biological processes such as sleep-wake cycles, blood pressure, core body temperature, hormone secretion, and energy metabolism are rhythmically fluctuate (3, 4). A disruption of circadian system, such as knockout of a circadian gene, altered light/dark cycle, shift work, and jet lag, contributes to obesity and its complications like hyperglycemia and insulin resistance, which brings a huge burden to health and economics (5–7).

Thermogenic fat, including brown adipose tissue (BAT) and beige adipose tissue, has a high capacity to burn fat and dissipate excess energy as heat to resist obesity and its related metabolic disorders (8). Adipose tissue containing a peripheral clock also shows a diurnal rhythm and is regulated by the circadian system. Brown fat exhibits thermogenic rhythms in nonshivering thermogenesis in C57BL/6J mice (9) despite the fact that such mouse strain is deficient in melatonin, which acts as an important effector of circadian clocks (10). Previous studies support that circadian disruption may lead to obesity by impeding BAT activity. For example, circadian disruption caused by prolonged daily light exposure leads to low activity in brown fat, which contributes to obesity (11). Mice fed on high-fat diet (HFD) gained more weight with the knockout of core clock gene brain and muscle arnt-like 1 (Bmal1) in BAT compared with the wild-type mice due to disrupted rhythms of fatty acid utilization and mildly reduced thermogenesis in BAT (12). Moreover, time-restricted feeding (TRF) can mitigate obesity by increasing rhythmic creatine-mediated thermogenesis (13). Hence, thermogenic fat may play an important role in obesity caused by circadian disruption, while the effect of circadian clock on thermogenic fat has not been fully elucidated. This review will focus on the role of circadian clock in the development and function of thermogenic fat, which might provide novel insights for the prevention and treatment of metabolic diseases by targeting thermogenic fat in a circadian manner.

## Thermogenic fat

Adipocytes in mammals include white adipocytes and thermogenic adipocytes. The white adipocytes are characterized by a large unilocular lipid droplet and few mitochondria. Thermogenic adipocytes have multilocular lipid droplets and large numbers of mitochondria expressing the mitochondrial protein uncoupling protein 1 (UCP1). Thermogenic fat is heterogeneous and consists of BAT and beige adipose tissue. BAT develops in the embryonic period and originates from Myf5^+^ lineage akin to skeletal muscle, which is mainly distributed in the interscapular region in rodents (14). Unlike BAT, beige adipocytes are derived from Myf5^-^ but PDGFRα^+^ non-dermomyotome cells postnatally and are recruited in white adipose tissue (WAT) depots, particularly in the inguinal region (also called browning process), in response to specific signals like cold and β3-adrenergic stimulants (15). Since beige adipocytes are recruited from white adipocytes, they are considered to be of an intermediate color between white and brown adipocytes. The main function of thermogenic fat is turning chemical fuel into physical heat, which is called non-shivering thermogenesis, to maintain the normal body temperature. Besides thermogenesis, thermogenic fat also participates in glucose, lipid, and amino acid metabolism, and thermogenic fat can resist adipose fibrosis (16). The PR domain containing 16 (PRDM16)-GTF2IRD1 complex in thermogenic fat can repress the transcription of TGF-β to inhibit pro-fibrosis genes and improve glucose homeostasis (17). Moreover, beige adipocytes have the ability to secrete β-hydroxybutyrate that acts on precursor cells to reduce fibrosis through the PRDM16-driven transcriptional signal (18). Activation of BAT and beige adipocytes can improve insulin sensitivity and combat obesity (8, 19–21). In recent years, non-shivering thermogenesis has become an attractive target for the therapy of obesity and associated metabolic disorders.

## Circadian rhythms in thermogenic fat

Circadian rhythms are present in thermogenic fat. Circadian genes and circadian-controlled genes show diurnal rhythms in BAT and iWAT (inguinal WAT) (22). Since body temperature shows circadian oscillations and affects many biochemical reactions, the temperature might be the original and universal resetting cue for circadian oscillators in mammals (23, 24). Animal studies suggest that the Ucp1 gene expression in BAT is rhythmical over a period of 24 h and that thermogenesis exhibits a circadian rhythm, which lead to rhythmic body temperature (9). The 24-h rhythm in glucose uptake by BAT is observed by ^18^F-FDG uptake imaging in mice (25). In addition, the synthesis of fatty acid shows high-amplitude circadian rhythms in thermogenic BAT during chronic cold in mice, while such rhythms are absent in thermoneutrality (26). Besides those animal studies, human studies also confirm the circadian rhythms in thermogenic fat. Studies suggest that BAT in adult humans shares a number of common features to beige fat (15, 27). Insulin-stimulated glucose uptake was also found to display a circadian rhythm in human BAT (28). In both mice and humans, fatty acid uptake by BAT shows strong rhythms at the onset of wakening, which may explain the rhythmic plasma lipid concentrations at waking (29). Similarly, another study involving healthy humans also confirmed that nonshivering thermogenesis and fat oxidation in BAT are more obvious in the morning than in the evening (30). Collectively, these studies show that thermogenic fat exhibits circadian rhythms in aspects of both genes and functions.

## Circadian disruption contributes to obesity

The central clock SCN and the peripheral clocks in peripheral tissues coordinate with each other in response to environmental cues, such as light, food, and sleep, to maintain circadian rhythms in almost all cells/tissues (1, 29). At the molecular level, the transcriptional–translational feedback loop (TTFL) is mainly involved in the cell-autonomous rhythms. The BMAL1 and Circadian Locomotor Output Cycles Kaput (CLOCK) comprise the key positive arm and bind to E-box sequences to promote the expression of Period (PER) and Cryptochrome (CRY), which then prevent the CLOCK : BMAL1 complexes from driving transcription further (1, 31). Additionally, the expression of nuclear receptors REV-ERB α/β and retinoic acid receptor-related orphan receptors RORs α/β are also driven by the CLOCK : BMAL1 complexes, and then the ROR, in turn, promotes the transcription of BMAL1, while REV-ERB suppresses transcription (32, 33). Thus, the TTFL is mainly composed of the positive arm including BMAL1, CLOCK, and ROR proteins and the negative arm containing PER, CRY, and REV-ERB proteins (31).

**Figure 1:**
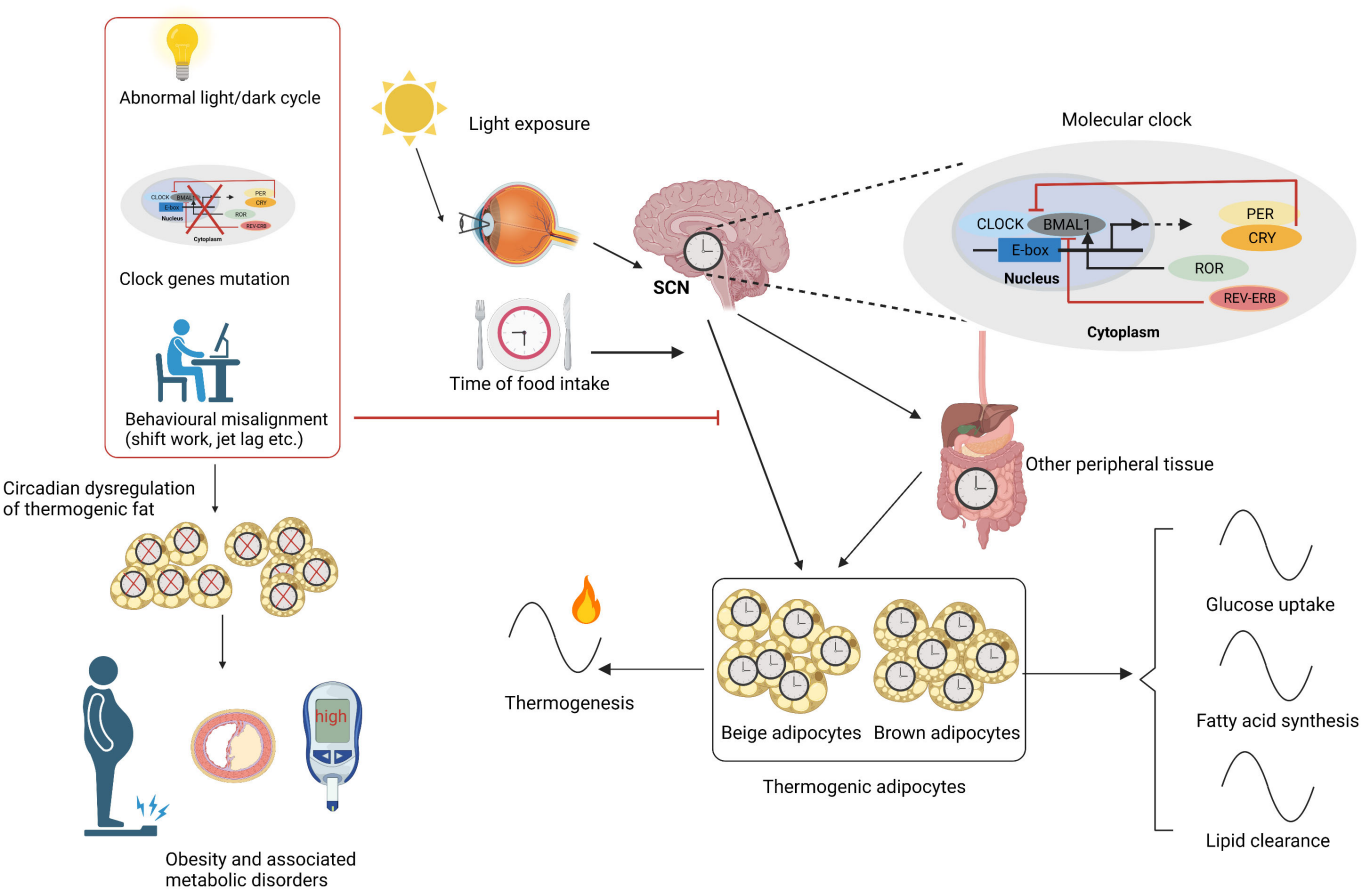
Circadian rhythms in thermogenic fat. SCN is mainly entrained by light signals and synchronizes the circadian rhythms of thermogenic fat and other peripheral tissues. SCN and other factors like time of food intake and peripheral tissues carefully regulate the thermogenic adipocyte clock. The molecular mechanism lies in TTFL including a positive arm such as CLOCK, BMAL1, and ROR and a negative arm like PER, CRY, and REV-ERB. Circadian rhythms including glucose uptake, fatty acid synthesis, lipid clearance, and thermogenesis are present in thermogenic fat. Circadian disruptions caused by abnormal light/dark cycle, clock gene mutation, and behavioral misalignments like shift work and jet lag cause circadian dysregulation of thermogenic fat, contributing to obesity and related metabolic disorders. SCN, suprachiasmatic nuclei; TTFL, transcriptional–translational feedback loop. Figures were created with BioRender (https://biorender.com/).

A number of biological processes, such as glucose and lipid homeostasis, energy expenditure, and hormone secretion, are regulated by the circadian clock (34, 35). The SCN is mainly entrained by light signals, and the peripheral clocks can be modulated by temperature, food, and sleep as well as hormonal cues (2). The abnormal expression of circadian genes, environmental misalignment like abnormal light/dark cycles, and behavioral misalignment, including feeding, sleep–wake cycles, and activity, can promote circadian disruption, which could contribute to obesity and obesity-related metabolic disorders (36, 37). For example, animals with a genetic circadian disruption, such as the mutation of CLOCK and the deletion of BMAL1, are prone to obesity and metabolic syndrome (38–40). Animals gained more weight under the altered light/dark cycle including shortened period and prolonged light as well as blue light (11, 41, 42). Behavioral misalignments, such as shift work, jet lag, and sleep disruption, can accelerate the occurrence and development of obesity and its associated metabolic disorders in both animal models and humans (7, 43–45), in which the inactivation of thermogenic fat may be involved (11, 13, 46, 47). The existence of circadian rhythms in thermogenic fat also suggested that it may participate in circadian disruption-associated obesity and relevant metabolic complications. The circadian rhythms in thermogenic fat and the related obesity under circadian disruption are shown in **Figure 1**. As thermogenic fat is becoming a promising target for treating obesity and metabolic disease, it is very important to figure out how thermogenic fat is regulated by the circadian clock to provide more accurate anti-obesity therapy.

## Circadian clock regulates the development of thermogenic fat

The development of brown and beige adipocytes is controlled by transcriptional regulation. Many clock genes encode transcription factors that impact adipogenesis and regulate the development of thermogenic fat. The deficiency of Bmal1 in both brown preadipocytes and mesenchymal precursors contributed to the formation of brown adipocytes by reducing TGF-β pathway activity and enhancing BMP signaling (48). The Bmal1-null mouse also had increased thermogenesis and adipogenesis in BAT (48).

Enhanced Ucp1 expression, more multilocular lipid droplets, and mitochondria were observed in the BAT and subcutaneous WAT in Rorα-deficient mice, and primary brown adipocytes with Rorα deficiency also showed a higher metabolic rate. Rorα deficiency promotes brown/beige adipogenesis by enhancing histone-lysine N-methyltransferase enzyme 1, which contributes to the development of thermogenic fat by stabilizing PRDM 16 transcriptional complex (49). Moreover, the RORα inverse-agonist SR3335 was confirmed to activate BAT and increase beige fat and adaptive thermogenesis in *in vivo* mice models and *in vitro* experiments. Human adipocytes also presented increasing beige characteristics after RORα inhibition, while the RORα agonist exerted an opposite effect (50). With respect to Rev-erbα, Gerhart-Hines et al. found that Rev-erbα could reduce thermogenic capacity by the transcriptional inhibition of Ucp1 in BAT so as to regulate circadian thermogenic plasticity in adult mice (9). However, Rev-erbα was found to promote BAT development in another study. The formation, structural integrity, and characteristics of BAT were impaired by the loss of Rev-erbα in neonatal mice. The Rev-erbα knockdown also diminished the brown fat-specific features in brown adipogenic differentiation, while its overexpression promoted brown adipogenesis by suppressing TGF-β signaling (51). The former study mainly focused on the role of Rev-erbα in Ucp1 expression levels in adult mice at thermoneutrality (9), while the latter study investigated its role in BAT development in neonatal mice at 22°C and *in vitro* experiments (51). The age of mice and ambient temperature may account for the differences in these two studies. The specific role of Rev-erbα in BAT development needs to be further explored by tissue-selective ablation models in future studies. Moreover, CRY1/2 was reported to increase brown fat-specific gene expressions and enhance brown adipocyte differentiation, in which the repression of BMAL1 and the interaction with PPARγ may be involved (52).

## Circadian clock controls the function of thermogenic fat

Early studies showed that excitation of the SCN by glutamate leads to an increase in BAT temperature (53). The SCN promotes fatty acid uptake from triglyceride-rich lipoproteins in the skeletal muscle as well as BAT, which maintains the day–night variations in plasma triglycerides (54). The diurnal rhythms are present in both thermogenesis and lipid clearance in BAT (9, 29). As a result, the function of thermogenic fat may be closely related to the circadian clock. As components of the TTFL, many key clock genes play vital roles in the function of thermogenic fat. The peroxisome-proliferator-activated receptor α (PPARα), as a distinctive marker of BAT, is widely considered to enhance lipid catabolism, activate the thermogenic function in BAT, and promote a white-to-beige conversion in WAT (55, 56). The CLOCK protein was also found to regulate the circadian expression of PPARα by combining its E-box-rich region (34). These studies suggest a possible role of CLOCK in the regulation of thermogenic fat.

The role of BMAL1 in thermogenic fat seems to be inconsistent in BAT. Global Bmal1-null mice had higher Ucp1 expression levels and could maintain core body temperature upon cold exposure, although having large lipid droplets (57). Another study also confirmed the increased thermogenesis and adipogenesis in BAT when Bmal1 was globally absent (48). The deficiency of BMAL1 in adipocyte was shown to contribute to obesity, although increased Ucp1 expression levels were observed (5). Moreover, the specific deletion of Bmal1 in BAT was also used to explore the relationship between BAT and thermogenic fat. The core body temperature was lower in brown adipocyte-selective Bmal1-deficient mice at 22°C, and the locomotor activity was similar compared with the control mice, while higher levels of thermogenic genes, such as Ucp1, Cidea, and Elovl3, were observed in BAT when Bmal1 was deficient (58). A recent study also found increased Ucp1 mRNA and UCP1 protein levels in BAT, with Bmal1 specifically deleted. Although the mice with Bmal1 KO in BAT had decreased UCP1-independent futile creatine cycling and mildly impaired thermogenesis, the cold tolerance was comparable to the control mice with increased shivering thermogenesis (12). The paradoxical results of the role of Bmal1 in core body temperature may be explained by the different Bmal1-floxed alleles (12, 58). REV-ERBα’s role in repressing Ucp1 may be involved in the effect of Bmal1 on Ucp1 expression levels (9). Recently, Xiong et al. reported that the overexpression of Bmal1 in beige fat inhibited beige adipogenesis and thermogenesis by regulating MRTF/SRF signaling in *in vivo* mice model. Correspondingly, the selective ablation of Bmal1 enhanced iWAT browning and improved glucose homeostasis (59).

Rev-erbα regulates circadian thermogenic plasticity by repressing Ucp1 gene expression (9). Additionally, during chronic cold exposure, circadian lipid synthesis in BAT was mediated by the circadian regulation of SREBP by Rev-erbα. Similarly, the specific absence of REV-ERBα in BAT led to increased Ucp1 after de-repression (26). Furthermore, Rorα-deficient mice induced increased thermogenic genes in BAT and iWAT, which might contribute to the resistance to diet-induced obesity (7, 60). Another study also confirmed that the deletion of RORα induced iWAT browning process by increasing the PGC-1A and PRDM16 levels in staggerer mice (61). However, the Per2 mutant mice were cold-sensitive due to impaired adaptive thermogenesis. PER2 could act as a co-activator of PPARα and upregulated fatty acid binding protein 3, which thus led to increased and activated Ucp1 (62).

Moreover, the factors regulating circadian rhythms also have an impact on the function of thermogenic fat—for example, as light is an important zeitgeber for SCN, constant light caused abnormal rhythms and reduced the UCP1 expression levels in BAT (63). Both constant light and dark conditions led to the absence of diet-induced thermogenesis in humans (64). Prolonged light decreased the uptake of fatty acids and the transcription of Ucp1 of BAT in mice (11). A study also reported that advanced light phase shifts led to a brown-to-white transformation and reduced the Ucp1 levels in BAT (65). Thermogenic fat is also regulated by the photoperiod with a short 8:16 light/dark cycle, causing higher expression levels of Ucp1 in both BAT and retroperitoneal WAT compared with the long 16:8 light/dark cycle in Siberian hamsters (66). Consistent with this, another study also confirmed that short photoperiod stimulated lipid mobilization and the browning of WAT (67). Opsin 3, a blue-light-responsive opsin, was found to enhance adaptive thermogenesis in mice through adipocyte light sensing (68). A more recent study figured out the neuroregulatory mechanism of light-modulating glucose metabolism in BAT (69).

The SCN is mainly entrained by light signals, while many peripheral clocks could be entrained by food signals (70). Temporally restricted food access entrained phase shifts of the circadian gene of BAT in mice (71). The impact of TRF under HFD was also explored in another study. HFD in mice disrupted the normal metabolic cycle, and TRF considered to restore rhythms increased the rhythmic Ucp1 and PPARα expression, which enhanced thermogenesis and resisted obesity (72). Time-restricted feeding during the inactive (light) period also increased thermogenesis in BAT and iWAT by controlling the adipocyte creatine metabolism in a circadian manner in mice (13). In a word, the abovementioned studies suggest that the functions of thermogenic fat, including thermogenesis and improvement of glucose and lipid metabolism, are all regulated by a circadian clock.

## Mechanism involving the circadian regulation of thermogenic fat

**Figure 2:**
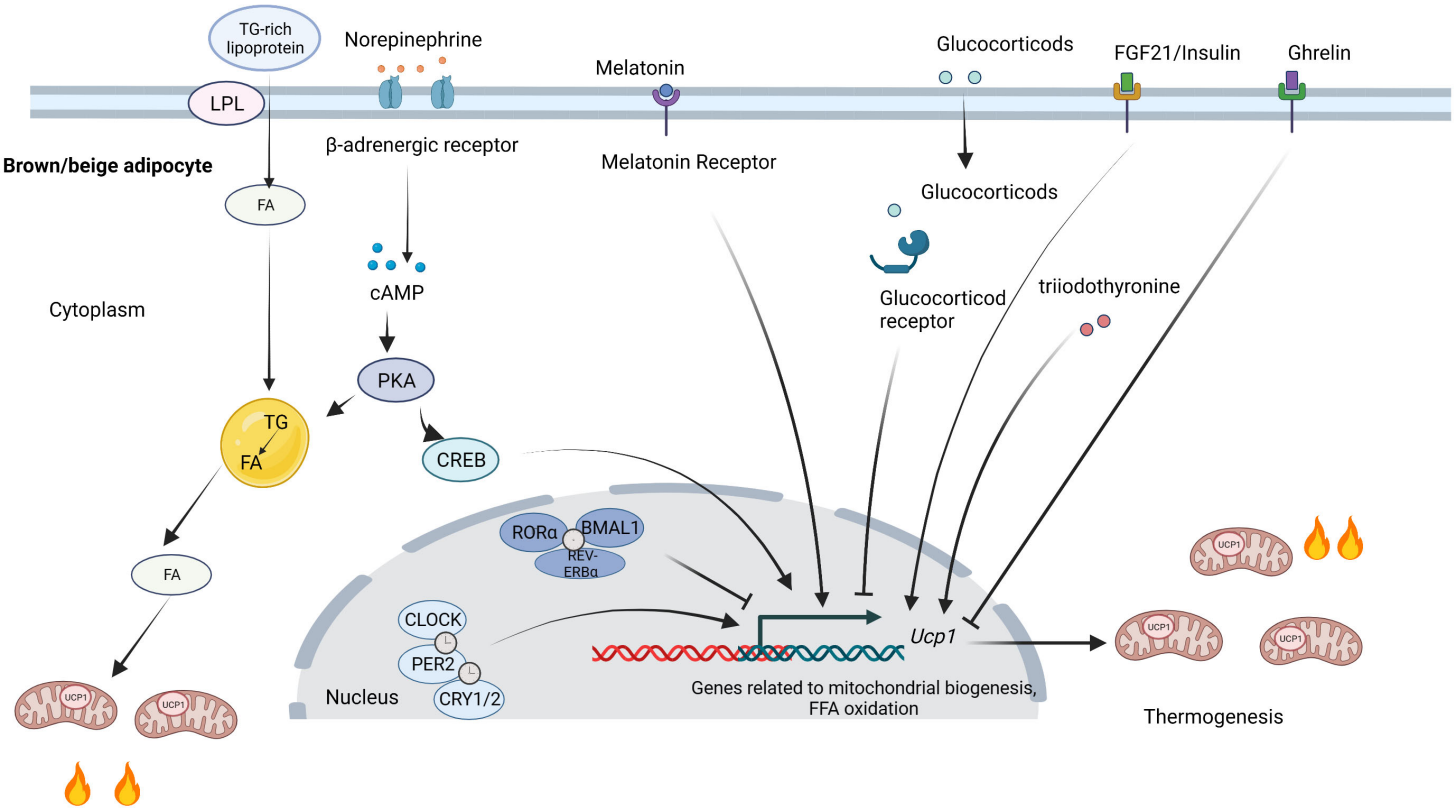
Circadian regulation of thermogenic fat. Clock proteins directly influence thermogenic fat function by regulating transcription. CLOCK, PER2, and CRY1/2 positively regulate thermogenesis, while RORα, BMAL1, and REV-ERBα inhibit thermogenesis. SNS activates thermogenic fat by releasing norepinephrine. FA released from lipid droplets can activate UCP1. The TG-rich lipoproteins promote FA uptake by LPL and replenish intracellular lipid stores. Melatonin, FGF21, and insulin bind to their receptors and contribute to thermogenesis, while glucocorticoids and ghrelin suppress thermogenesis. SNS, sympathetic nervous system; TG, triglyceride; FA, fatty acids; UCP1, uncoupling protein 1; LPL, lipoprotein lipase; FGF21; fibroblast growth factor 21. Figures were created with BioRender (https://biorender.com/).

The factors regulating circadian rhythms also influence thermogenic fat function, while the exact mechanism remains to be illuminated. β3-adrenergic signaling is a classic beiging signal that induces adaptive thermogenesis in brown and beige adipocytes by promoting cAMP production in mice (73, 74). In addition, many hormones vary across the day and night and participate in the regulation of thermogenic fat (75, 76). Most clock genes, as transcription factors, directly regulate the transcription of thermogenic genes and genes involving glucose and lipid metabolism as mentioned above. Besides the direct effect of clock genes, circadian sympathetic innervation and hormones are also involved in the circadian regulation of thermogenic fat. The circadian regulation of thermogenic fat is shown in **Figure 2**.

### Circadian regulation of thermogenic fat by the sympathetic nervous system

The activity of thermogenic fat is largely controlled by the sympathetic nervous system (SNS). There are neuroanatomical connections between SCN and BAT, and SCN neurons cooperate with other neurons in controlling sympathetic activity in BAT (77). Glutamate injection into the SCN enhanced BAT thermogenesis and increased the core temperature in rats, which was mediated by the ipsilateral ventromedial hypothalamic (VMH) nucleus (53). Light inputs entrain the circadian clocks *via* the light-sensitive ganglion cells in the retina, and disruption of the light–dark cycles causes the inactivation of thermogenic fat. The reduced β3-adrenergic intracellular signaling input accounts for the impaired BAT activity in mice under prolonged daily light (11). The uptake of fatty acid in BAT presents circadian rhythms, which were lost after the sympathetic denervation of BAT (29). A short photoperiod could resist obesity in Siberian hamsters by increasing SNS-stimulated lipid mobilization and inducing WAT browning (67). Interestingly, Meng et al. recently found that hypothalamic supraoptic nucleus (SON) collects light signals by intrinsically photosensitive retinal ganglion cells and then passes signals to paraventricular nucleus neurons, which caused the activated GABAergic neurons in the solitary tract nucleus. This process finally led to impaired adaptive thermogenesis in BAT and decreased glucose tolerance through β3-adrenergic signaling. This study revealed a novel neural circuit of SON modulating light-mediated glucose tolerance independent of SCN (69). These results indicate that light signals may modulate lipid metabolism and thermogenic activity in thermogenic fat by SNS.

Besides light signals, Orozco-Solis et al. investigated how nutrition signals regulate circadian energetics. As VMH is involved in nutrient sensing, the deletion of core-clock gene Bmal1 specifically in Sf1-neurons of the VMH resulted in increased energy expenditure and enhanced thermogenic capacity mainly in BAT *via* adrenergic signaling, which suggested that the VMH clock can regulate circadian thermogenesis independent of the SCN and the endogenous BAT clock by receiving inputs from environmental zeitgebers (78).

However, Razzoli et al. reported that mice lacking β-adrenergic receptors maintained the circadian rhythmicity of Ucp1 and clock genes of BAT despite the low Ucp1 expressions at room temperature and cold challenge. This indicates that β-adrenergic receptors modulated Ucp1 during cold challenge without influencing its circadian rhythmicity (79). Similarly, thermogenesis still exhibited circadian rhythms at thermoneutrality when the sympathetic outflow to BAT was minimal (9). These further confirm that other factors, like clock genes and the following hormones, also participate in the circadian regulation of thermogenic fat apart from circadian sympathetic innervation.

### Circadian regulation of thermogenic fat by hormones

When referring to the circadian releases of hormones, melatonin is one of the most important circadian hormones as it rises as light fades and peaks during darkness (80). The circadian rhythms of melatonin are produced by the pineal gland under the control of SCN. Circadian disruptions like abnormal light, shift work, and jet lag could lead to impaired rhythms of melatonin (80). Even low-intensity light like LED in the evening can delay the phase and reduce melatonin secretion (81). Besides in the brain, melatonin receptors were also observed in adipocytes (82). Melatonin has been shown to promote circadian rhythm-mediated proliferation in WAT (83). The association between melatonin and thermogenic fat has also been reported. Ryu et al. reported that a short photoperiod increased the iWAT browning and BAT activity and thus reversed obesity compared with a long photoperiod, in which melatonin was involved in stimulating a sympathetic activity (67). In addition, pinealectomized rats with melatonin absence were overweight with reduced UCP1 levels in BAT and showed intolerance to cold, which was reversed by melatonin treatment (84). Melatonin is also reported to induce iWAT browning and promote BAT activity in other studies, while the exact mechanism remains to be investigated, and the increased sympathetic drive by SCN and the direct effects of its receptors in fat might be involved (85–88). In view of the role of melatonin in circadian rhythms and thermogenic fat, melatonin is considered to participate in the circadian regulation of thermogenic fat.

Glucocorticoids also show robust daily variation under the regulation of the hypothalamus–pituitary–adrenal gland. The rhythmic secretion of glucocorticoid is controlled by SCN as the diurnal rhythms were completely blunted after the destruction of the SCN (89). Many clock proteins can regulate the activity of glucocorticoids. CLOCK can directly repress the transcriptional activity of the glucocorticoid receptor (GR) (90). Another study showed that CRYs mediated the rhythmic repression of GRs (91). In turn, glucocorticoid signaling can also reset the phase of circadian time in peripheral tissues (92). Many clock genes like *Per1* and *Per2* that contain GR-responsive elements can be regulated by glucocorticoids (93). Blue light increased the expression of circadian genes in the WAT partly by increasing plasma corticosterone (42). The constant light causes blunted corticosterone rhythm in mice (94). These further confirm the close relationship between circadian rhythms and glucocorticoids. Genes associated with BAT function are also modulated by glucocorticoids in a GR-dependent fashion (95). Glucocorticoid signaling activation has been shown to inhibit BAT thermogenesis and induce the whitening of beige adipocytes (96, 97). The disruption of glucocorticoid diurnal rhythms could lead to the dysregulation of thermogenic fat—for example, the diurnal range of corticosterone concentration increases lipid storage and suppresses nonshivering thermogenesis in BAT (98). The flattening of circadian glucocorticoid oscillations similar to jet lag causes adipocyte hypertrophy and reduced UCP1 levels in BAT (99). Moreover, another study also reported that flattened corticosterone rhythm led to the loss of circadian rhythm in BAT-mediate triglyceride-derived fatty acid uptake possibly by reducing sympathetic innervation (100). As glucocorticoid is tightly regulated by circadian rhythms and its dysregulation can impair thermogenic fat function, the circadian regulation of thermogenic fat is at least partly mediated by glucocorticoid.

Apart from melatonin and glucocorticoid, some other hormones are also involved in the circadian regulation of thermogenic fat. Mistimed feeding was shown to disrupt the rhythms of serum melatonin, fibroblast growth factor 21 (FGF21), and ghrelin in growing pigs (101). FGF21, mainly secreted by the liver, exhibits a circadian oscillation, and its induction can activate thermogenesis in BAT and increase iWAT browning (102–104). The circadian expression levels of FGF21 can be regulated by clock genes such as REV-ERBα and RORα (105, 106). FGF21 signaling also feeds back SCN and regulates circadian behavior (107), which may indicate its circadian regulation in thermogenic fat. Furthermore, ghrelin, as a hunger-inducing hormone, also shows daily oscillations (108, 109). Circadian misalignment by a 12-h behavioral cycle inversion elevated the postprandial ghrelin levels (110). Ghrelin has been shown to inhibit BAT function by noradrenaline, and the suppression of ghrelin receptors activates BAT function (111, 112). Moreover, insulin and triiodothyronine are considered to enhance thermogenic fat activity (113, 114) and present circadian rhythms under the regulation of the circadian clock (115). Despite the fact that the studies about these abovementioned hormones and the circadian regulation of thermogenic fat are relatively scarce, they may be more or less involved in the regulation of thermogenic fat through circadian rhythmicity. Further investigations are needed to explore the exact associations and mechanisms.

## Conclusion and perspective

Collectively, almost all organs and tissues are under the control of circadian clock to adapt to environmental changes. Lots of cases of obesity and its associated diseases are accelerated by circadian disruption such as clock deficiency, prolonged light, and other ways. Like most other tissues, thermogenic fat shows strong circadian oscillation by a complex interplay of local clocks and a central clock. Circadian factors like light, feeding, and clock genes have an impact on the thermogenic clock. The differentiation and development of thermogenic fat are well orchestrated by a circadian clock. Most aspects of thermogenic fat function, including thermogenesis and glucose and lipid metabolism, are all under circadian control to promote metabolic homeostasis. The adipocyte clock genes, SNS, and some rhythmic hormones appear to be involved in the circadian regulation of thermogenic fat.

Several questions involving the circadian clock and thermogenic fat still remain. Firstly, the association between BAT and circadian rhythms has been reported in many previous studies, while studies on beige fat are relatively few. The late discovery of beige fat may partly explain this, and more research need to be made. Secondly, despite the role of clock genes, SNS, and some hormones that have been found to participate in the circadian modulation of thermogenic fat, other unknown mechanisms also need to be explored as circadian regulation is coordinated by many molecules both in transcription and posttranslational modification levels. Moreover, emerging studies also show that human BAT exhibits circadian rhythms. Chronotherapy targeting thermogenic fat like TRF seems promising, while relevant studies are not enough, especially in humans. Further studies are needed to resist obesity and metabolic disorders by therapeutically targeting circadian rhythms in thermogenic fat. Lastly, the exact contribution of dysfunction in thermogenic fat to the obesity phenotype is unclear and difficult to explain. A deeper understanding of the extent and exact mechanisms may offer novel avenues for combating metabolic disease.

## Author contributions

XP and YC wrote the manuscript. YC edited the manuscript. All authors contributed to the article and approved the submitted version.
